# The Unmasking Effect: Propofol-Induced Brugada Pattern in a Critically Ill Patient

**DOI:** 10.1155/2022/9226861

**Published:** 2022-06-08

**Authors:** Esiemoghie Akhigbe, Ebubechukwu Ezeh, Benjamin Dao, Kelechukwu Okoro, Paul Okhumale

**Affiliations:** ^1^Department of Internal Medicine, Marshall University Joan C. Edwards School of Medicine, Huntington, WV, USA; ^2^Department of Cardiovascular Services, Marshall University Joan C. Edwards School of Medicine, Huntington, WV, USA

## Abstract

Brugada syndrome is a known cause of dysrhythmias and sudden cardiac death. It is linked to mutations in myocardial sodium channel leading to hyperexcitable cardiac myocytes. The use of this sedative has been linked to the development of inducible Brugada via blockade of sodium currents in cardiac myocytes. Although propofol is usually avoided in patients with known Brugada syndrome, some patients might have undiagnosed Brugada syndrome and thus are at risk for complications. We present a case of propofol induced Brugada in a critically ill patient.

## 1. Introduction

Brugada syndrome (BrS) is an autosomal dominant genetic disorder that is associated with malignant arrhythmias and sudden cardiac death [1, 2]. The enhanced myocardial excitability is largely attributed to mutations in the myocardial sodium channel. Due to antagonization of sodium channels, propofol's use as an induction and sedation agent in patients with BrS has traditionally been avoided. In fact, the BrugadaDrugs.org Advisory Board recommends avoiding administration of propofol in patients with Brugada syndrome [3]. However, recent case reports and case studies have described the safe use of propofol without arrhythmic sequelae [4]. We describe a case of unmasked type I Brugada pattern electrocardiogram (EKG) due to prolonged propofol infusion in a critically ill patient.

## 2. Case Presentation

Patient is a 35-year-old man with no known past medical history presented from an outside facility on account of with epigastric abdominal pain, nausea, and vomiting of 3 days duration. He was subsequently diagnosed with gallstone pancreatitis for which he was taken to the operating room for a laparoscopic cholecystectomy. Patient was induced with propofol and intubated perioperatively but aspirated during intubation. During the laparoscopy, the patient was noted to have dilated small bowel loops, and the cholecystectomy was terminated. The patient was extubated postoperatively. A follow-up computed tomography (CT) scan demonstrated necrotizing pancreatitis, and the patient was transferred to our facility.

**Figure 1:**
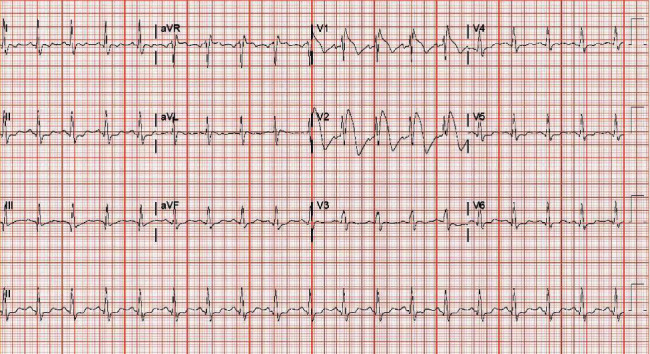
Electrocardiogram on day 12 of admission showing characteristic type 1 coved shaped ST segment elevation in leads V1 and V2.

**Figure 2:**
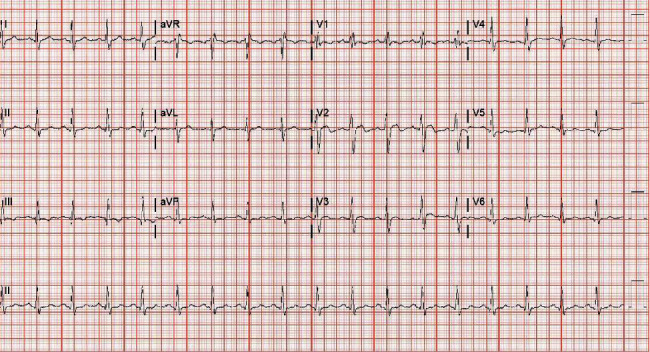
EKG 10 hours following cessation of propofol administration showing reversal of Brugada pattern.

After arrival at our facility, the patient was again induced with propofol for cholecystectomy. The patient was noted to have dense intra-abdominal adhesions necessitating the need for an open cholecystectomy. The patient tolerated the procedure well and was extubated. On postoperative day (POD) 3, the patient developed acute respiratory failure secondary to hypovolemic shock. He was reintubated and again induced with propofol. Patient was later found to have a pancreatic hematoma with no active bleeding and was treated medically. Patient postoperative period was also complicated by an extensive pulmonary embolus for which he underwent pulmonary artery thrombectomy and inferior vena cava filter placement after which he remained intubated and sedated. On POD 11, patient developed abdominal distension with anuria and was taken back to the OR where a decompressive exploratory laparotomy with evacuation of an intra-abdominal hematoma was performed. On POD 12, an EKG was obtained and demonstrated type I Brugada pattern (Figure 1). After identification of type I Brugada pattern, propofol was discontinued, and EKG changes resolved within 10 hours (Figure 2). The patient received a total of 12880 mg of propofol (Table 1). The patient had a prolonged hospital course that lasted a total of 83 days and underwent other multiple interventions. The remainder of hospitalization was not complicated by any cardiac arrest or malignant arrhythmia.

## 3. Discussion

Brugada syndrome (BrS) is an autosomal dominant channelopathy associated with malignant arrhythmias and sudden cardiac death [2]. It is known to have variable penetrance with a strong male predominance [5]. BrS has been linked to a myriad of etiologies, but it is widely associated with mutations in the genes encoding the cardiac sodium channels. The genes implicated are the SCN5 and SCN10A. Mutations in this channel lead to an unopposed potassium current into the epicardium leading to its instability. Various hypotheses have been used to explain the ECG changes in BrS. These include (a) the repolarization hypothesis, involving the presence of a voltage gradient due to transmural or transregional dispersion of the action potential of different layers of the right ventricle at the beginning of repolarization, as a consequence of a loss of Na current combined with a dominant transient outward K current (Ito) and (b) the depolarization hypothesis, involving right ventricular conduction delay at the end of depolarization combined probably with subtle structural right ventricular anomalies [5].

BrS is characterized by distinct ST segment changes with appearance of a right bundle branch pattern and ST segment elevation in leads V1-V3 coupled with either sudden cardiac arrest, sustained ventricular arrhythmia, atrial fibrillation, nocturnal agonal respiration, or symptoms suggestive of ventricular arrhythmias. [6]

Brugada pattern is the presentation of EKG findings without clinical features. Like BrS, It is subclassified into three types depending on phenotypic appearance on the EKG (Table 2). Type I Brugada pattern is characterized by coved ST-segments in V1 and V2, and type II Brugada pattern is characterized by saddle-back ST segments where the ST segment descends before rising again to an upright or biphasic T wave, while type III is a combination of both types 1 and 2 without satisfying the criteria for either type 1 or 2 [5–7]. Multiple factors have been identified to precipitate BrS including fever, sodium channel blockers, anesthetics, tricyclic antidepressants, and cocaine [8]. Drug-induced Brugada syndrome from noncardiac drugs occurs predominantly in adult males and is frequently due to drug toxicity [9].

Propofol is a widely used sedative-hypnotic in surgeries and in the management of critically ill patients. Since its widespread implementation, propofol infusion syndrome (PRIS) has been identified, which consists of metabolic acidosis, rhabdomyolysis, hyperkalemia, and sudden cardiac death. These sudden cardiac deaths have been attributed to malignant arrhythmias. Propofol has been demonstrated to have sodium channel blocking properties. Multiple case reports have identified Brugada patterns in patients with prolonged propofol infusion and/or induction [10]. It is currently recommended to avoid propofol in patients with BrS to prevent inducing malignant arrhythmia [3]. Although the exact mechanism in which propofol induces BrS is unknown, the resolution of EKG findings in the patient following discontinuation of the medication points to a possible role of propofol in arrhythmogenesis.

Multiple case studies have, however, demonstrated a Brugada type EKG change heralding ventricular arrhythmia or sudden cardiac arrest. Junttila et al. demonstrated with their case series that Brugada type EKG changes are at higher risk for sudden cardiac arrest [11]. It has been demonstrated that EKG changes associated with propofol-related infusion syndrome is a sign of electrical instability and can herald cardiac arrest [12].

There have been multiple other cases describing propofol-induced Brugada pattern EKG changes, but these were in the context of PRIS. Although this patient did not have any other signs of PRIS, our patient demonstrated Brugada-type EKG changes secondary to prolonged propofol infusion. Cardiac arrest or sudden cardiac death were avoided by discontinuation of propofol infusion.

### 3.1. Follow-Up

Patient was scheduled for outpatient follow-up upon discharge for genetic testing and repeat EKG. However, patient was lost to follow-up.

## 4. Conclusions

Propofol has traditionally been avoided in patients with BrS. Although recently, there have been multiple reports of patients with Brugada syndrome that have safely tolerated propofol infusion or induction without any arrhythmia. This case demonstrates that sudden cardiac death, cardiac death, or malignant arrhythmia can be avoided in propofol-induced type I Brugada pattern with discontinuation and avoidance of propofol.

## Figures and Tables

**Table 1:** Table showing total amount of propofol received by patient during hospital course.

POD	Propofol dose (mg)
1	180
2	20
3	1070
4	1430
5	1680
6	1440
7	1680
8	1680
9	1680
10	1480
11	540

**Table 2:** Patterns of ST abnormalities in leads V1-V3.

Feature	Type 1	Type 2	Type 3
*J* wave amplitude	≥2 mm	≥2 mm	≥2 mm
*T* wave	Negative	Positive or biphasic	Negative, positive, or biphasic
ST-T configuration	Coved	Saddle-back	Coved or saddle-back
Terminal portion of ST segment	Gradually descending	Elevated ≥1 mm	Gradually descending or elevated ≥1 mm

## Abbreviations

BrS: Brugada syndrome
EKG: Electrocardiogram
CT: Computerized tomography
POD: Postoperative day.
